# Lived experiences and informed behavior change among premenopausal women in a group exercise trial: a qualitative inquiry

**DOI:** 10.3389/fragi.2025.1712679

**Published:** 2025-11-26

**Authors:** Jaclyn Inel Hadfield, Ryan Hulteen, Jenna Parsons, Heather Allaway

**Affiliations:** School of Kinesiology, Louisiana State University, Baton Rouge, LA, United States

**Keywords:** women, premenopausal, group exercise, health behavior, qualitative inquiry

## Abstract

**Introduction:**

Before menopause, women experience declines in physical activity, which is a gendered phenomenon. This study explores factors that inform physical activity beliefs, behaviors, and experiences among premenopausal women participating in a group-based dance fitness workplace intervention.

**Methods:**

Two focus groups (n = 11) were conducted with participants enrolled in a 6-week group-based dance fitness trial. A narrative inquiry using thematic analysis was used to identify themes to contextualize the experience participants had within the context of the intervention.

**Results:**

There were lived experiences that informed participants’ intervention experience, namely, a desire for exercise consistency and a historically negative relationship with exercise. Women consistently reported that the exercise done in the intervention was fun and brought feelings of social connectedness and comfort, thus positively impacting their experiences. These encounters led to the reporting of changes in behavior, perceived health outcomes, and perceptions of exercise, which positively influenced their intention to continue being physically active.

**Conclusion:**

This study provides an understanding of intervention strategies and lived experiences that may be useful to improve the physical activity behaviors and health of women before menopause occurs. Physical activity programs incorporating women’s lived experiences and unique preferences may align with the exercise women report a desire to have in their lives.

## Introduction

Menopause is a life event where women experience significant health changes. The average age of menopause onset is 51 (Gold et al., 2001). Two million women reach menopause in the United States annually (6,000 per day), and these women will spend up to 40% of their lives in a postmenopausal state (Menopause, 2024). Participation in physical activity (PA) is recommended for women throughout the lifespan to prevent adverse health outcomes associated with menopause, like osteoporosis (Grindler and Santoro, 2015). Still, women experience PA declines before menopause, which are then exacerbated during the menopausal transition (Troiano et al., 2008). Moreover, women participate in less PA than men globally and throughout the lifespan (Elgaddal et al., 2022; Kohl et al., 2012; Troiano et al., 2008). This is a health disparity.

PA engagement is a gendered phenomenon with differences in women’s and men’s exercise preferences and behaviors. For example, dance fitness programs are dominated by female participants and are one of the top preferred forms of group exercise modalities among women (Exercise Movement and Dance Partnership, 2022). Dance fitness can take many forms (i.e., provides variety), requires little to no equipment, and consists predominantly of aerobic activity (a key part of PA guidelines (U.S. Department of Health and Human Services, 2018)). These factors make dance fitness an accessible and suitable lifelong PA behavior for women (Hulteen et al., 2015). Dance fitness typically incorporates full-body cardio-based movements and emphasizes enjoying oneself. Still, it differs from a traditional dance class in that it does not teach dance technique or choreography practices. Meta-analytic evidence of dance interventions further demonstrates its promise as an intervention modality to improve women’s health (Yan et al., 2024). Many forms of PA have been shown to improve health outcomes (Noetel et al., 2024). However, fewer studies have tried to account for the types of PA behaviors explicitly tailored to women’s needs and interests (Anderson et al., 2023; Exercise Movement and Dance Partnership, 2022; Segar et al., 2002).

Health professionals need to understand more about the gendered experience within PA to address women’s sustained behavioral engagement with PA across the lifespan. Specifically, there is a need to improve the PA levels and health of women between the ages of 30 and 60 years old. This age range encapsulates midlife adulthood and is associated with several new roles and life transitions for women (i.e., childbirth, menopause, career changes). Thus, practical strategies for promoting PA and health before menopause are needed to safeguard women’s health behaviors and outcomes. Data that contextualizes the gendered nature of PA can better inform intervention designs to positively influence women’s behavioral engagement with PA to promote overall health.

Most women of all age groups do not meet the PA guidelines, which have been proven to prevent disease and improve overall health (CDC, 2022). Nevertheless, one of the consistently top-trending topics online is fitness among women (Vega, 2019). We argue that the current content and delivery of women’s exercise is not equitably serving the behavioral needs of women’s health. In a recent poll, 63.9% of women reported having an interest in doing fitness classes, but only 17.3% of them engaged in these classes (Vega, 2019). It is important to consider that there are barriers in place keeping women from engaging in exercise behaviors that interest them.

Qualitative inquiry allows for more meaning to be extrapolated in understanding the nuanced relationship women have with PA and its connection to their health. As such, we qualitatively explored the experiential characteristics of premenopausal women participating in a group-based dance fitness workplace intervention to identify effective strategies for promoting PA and health among this population. This study specifically explores the question: What factors inform PA beliefs, behaviors, and experiences among premenopausal women enrolled in the Women Exercising, Active, and Learning Together for Health (WEALTH) workplace intervention trial?

## Methods

### Design

Our qualitative focus group study was a sub-study within a single-arm feasibility trial. The trial was undertaken at a southern university campus in the United States. Ethics approval for the present study was obtained from the university’s institutional review board (IRBAM-24-0049).

### Setting

The study occurred on a college campus in the southern United States.

### Participants

Participants included in this study met the inclusion criteria if they were: a) between the ages of 30 and 60, b) had at least one self-reported menstrual period in the last 3 months, and c) self-reported not currently meeting aerobic PA guidelines (i.e., less than 150 min of moderate-to-vigorous PA). Exclusion criteria included: a) younger than 30 or older than 60, b) self-identifying as male, c) exceeding current aerobic PA guidelines, and d) not being employed at the university campus where the intervention was held. All participants in the present study were enrolled in the single-arm WEALTH feasibility trial. Eleven premenopausal women participated in the study (mean age: 39.75 years ± 6.06).

### Description

A non-randomized, single-arm feasibility trial was conducted with full-time women employees at the southern university campus in the United States. Intervention programming (i.e., dance fitness group classes) was given twice a week, for 30 min per session, across 6 weeks in the spring semester (March and April 2024). Programming occurred during the typical lunch period from 12:15 to 12:45 p.m. This was considered the most feasible time for a PA intervention embedded within the traditional workday. All classes were led by a certified group fitness instructor with over 15 years of experience teaching dance and fitness classes. Classes were held in the Dance Studio housed within the School of Kinesiology on the US southern university campus.

### Recruitment

Participants were recruited to the main study’s intervention through a variety of modalities including, but not limited to, e-mail, posted flyers around campus, social media posts from university accounts, public advertisements through university media channels, or in person contact via word of mouth. All materials had either a QR code or hyperlink that took individuals to an information letter to learn more about the intervention and main study. Participants were then able to decide to proceed and view a consent form, where formal enrollment in the study occurred. Table 1 provides sample demographic characteristics of all women enrolled in the main study intervention.

**TABLE 1:** Descriptive characteristics of study sample (N = 28).

Age	39.75 (6.06) years	Individual income	
Race		35,000–55,000	6
White	19	55,001–75,000	13
Black/African American	7	75,001–95,000	3
Other	1	95,001+	4
No response	1	No response	2
Hispanic, latino, or Spanish origin		Sexual orientation	
Yes	1	Heterosexual	24
No	25	Prefer not to answer	3
No response	2	No response	1
Highest education attained		Staff/Faculty status	
High school diploma	1	Faculty	8
Trader certificate/Diploma	1	Staff	19
College/University certificate or diploma	1	No response	1
Bachelor’s degree	6	Work status	
Master’s degree	14	Full time	27
PhD	4	Part time	0
No response	1	No response	1

Participants in this paper’s qualitative study were recruited to participate in the study during the intervention by advertising the focus group opportunity before and after weekly classes. Recruitment efforts consisted of emails to all enrolled intervention participants, and announcements before and after the class. Those that were interested were prompted to email the primary investigator of the study to official enroll in the qualitative study. These participants were then scheduled to participate in a focus group during the last week of the intervention trial.

### Focus groups

Two focus groups (n = 11) were conducted. Each focus group was conducted in the dance studio after the WEALTH class finished. The instructor was not present in the studio to ensure participants could express their responses to a neutral interviewer, a research assistant. To clarify, the interviewer was a co-author. While the role as a co-author may imply familiarity with the study context, their position as a research assistant was intended to serve as an impartial data collector to minimize bias. We acknowledge that their co-author status could be perceived as reducing neutrality, and acknowledge this to ensure transparency.

Participants were familiar with one another from participating in the trial and shared similar characteristics due to all meeting the inclusion criteria, which fostered group cohesion. A research assistant with training in qualitative inquiry led the focus group. Again, this was intentional to address possible with issues with bias since the participants did not have a connection or familiarity with this research assistant and the study. Each focus group discussion was recorded and transcribed. The focus group guide (see supplemental materials) was tailored to extrapolate aspects of the intervention delivery and participants’ exposure to the intervention that influenced participants’ attitudes, behaviors, and experiences related to their exercise engagement.

### Analysis

A narrative inquiry using thematic analysis was used to identify themes to contextualize the experience participants had within the context of the intervention. It has long been a goal of narrative inquiry to link personal experience with cultural knowledge, ideology, and grand or master narratives (Riessman, 2008). As such, this lens of inquiry was most appropriate in extrapolating data that could speak more broadly to the cultural understanding of exercise as the underpinnings in addressing our aim. Narrative inquiry allows researchers to gather data on participants’ perspectives through their lived experiences. We used open-ended questions in the focus groups to avoid leading the participants. The thematic analysis then allowed us to organize patterns in participants’ narratives and their connections to attitudes, behaviors, and experiences within the context of the exercise intervention. Thematic analysis can orient experientially to the data, hence its utility in our study (Braun and Clarke, 2021). We went through the general steps when conducting thematic analysis: becoming familiar with the data, coding, generating first round themes, reviewing themes, refining and defining themes, report write-up. All these steps were guided by our research question: What factors inform PA beliefs, behaviors, and experiences among premenopausal women enrolled in the Women Exercising, Active, and Learning Together for Health (WEALTH) workplace intervention trial? We refined themes with this question in mind and defined the following themes that each connected to beliefs, behaviors and experience within the context of our research question and aim: (1) predetermined experiences, (2) intervention design factors, and (3) actual changes.

## Results

We qualitatively explored the experiential characteristics of eleven premenopausal women in the WEALTH trial. Our analysis highlighted the following primary structural categories: (1) predetermined experiences, (2) intervention design factors, and (3) actual changes. These categories organized participants’ implied PA experiences, beliefs, and behaviors. We present our findings based on these structures to show how participants contextualize a relationship with exercise through the WEALTH trial.

### Predetermined experiences

These experiences were categorized as events that shaped and predetermined women’s relationship with exercise. There were lived experiences women brought with them into the intervention that informed their experience, namely, a desire for exercise consistency and a historically negative relationship with exercise. One participant described herself as having a *“love-hate”* relationship with exercise. While another empathized with her and shared, *“[exercise] feels obligatory sometimes.”* There was a group understanding of having experienced a negative relationship with exercise at some point. *“I do find out sometimes, like I have a negative relationship with exercise, where I’ll judge myself if it's been too long. It's really hard to get back into it.”*

We then noted how these negative experiences were connected to a desire for consistency with exercise. Some shared that they felt good when they were consistent with exercise, but could never remain consistent. Some women shared how they wanted consistency now because they *“used to be in better shape”* and felt like not having current consistent exercise was the cause of this perceived physical change. One woman questioned her meaning of exercise while still describing the observed connection between consistency and exercise.

“I do not consider myself active because I don't have like a regular routine, besides running behind two children all the time and picking up stuff … but somehow I associate exercise with seeing changes or results to you. Whereas activity doesn't necessarily mean that … like I can be outside gardening and I’m like lifting and moving and all that, but exercise is associated with, hey, I want an outcome … but I want to be more consistent with doing it.”

Another participant described how she has historically seen exercise as unfavorable throughout her life, but having had a consistent and positive exercise routine with WEALTH challenged that predetermined belief rooted in her past experience with exercise.

“I keep on forgetting that exercise is good for you. Like, I know it's good for you, and it's a necessary thing, but it can be fun. And so like [WEALTH], knowing that this was a treat for me to be able to come to this dance class.”

### Intervention design factors

Participants’ lived experiences, in tandem with intervention design factors, influenced their personal experience in the intervention. Through the definition of this structural category we were able to further identify subthemes to specify the actual design factors that impacted experiences. Women reported design factors that brought feelings of fun, comfort, and connection. These reported factors served as subthemes. Collectively, these factors and their influence on beliefs positively informed their experiences.

#### Fun

The nature of the dance fitness modality was a feature that prompted feelings of fun. These feelings were often conjured with associations of the dance moves used and how women moved their bodies in the intervention.

“The laughter like just being able to look over at a colleague for a certain dance move and just laugh … It’s fun. How silly you looked at the beginning.”

“This has been so fun and like such a good way to see faces of people that are in the building or not in the building. And it's just been very fun, and I feel so good after every one.”

One participant shared how the fun nature of the intervention changed her beliefs about exercise.

“… this was exciting for me to come to you. So I had fun. It made me realize that exercise doesn't have to be inside of a gym. I associate it with going to a facility, getting on a treadmill, and my self image problems would often get in the way of me doing that, So I realized that I don't have to be inside a gym on a treadmill to exercise.”

#### Comfort

Participants shared how the intervention made them feel comfortable and how this feeling positively informed their experience. Most participants felt comfort because the intervention was only for women. *“All women’s class … I think you are less like worried about making mistakes. I mean, this was a really laid back group. And so it was very accepting when nobody cared.”* There was a connection between feeling comfortable and exercising among only women for most participants.

“I feel more comfortable and I’m more motivated to work out when I’m around people, especially when it's an all women situation. I found that throughout my life, when I’m most consistent is when there’s a group of women that are working out with me.”

“Like getting to dance with a group of other women. I usually do YouTube videos by myself at the house, and it's just nice to be able to be in a studio with a mirror and other people doing the same dance as I.”

“The main thing, even I feel like just sometimes as a woman, even if a man is not doing anything to make me feel uncomfortable or judged, I just feel more comfortable around women in tennis shoes or forgot my shirt or whatever.”

There were also objective intervention factors that raised feelings of comfort for participants. Some reported feelings of comfort with the intervention being a movement modality that was accessible to various levels of fitness. *“It was like very accessible regardless of your like fitness level. There were moderations for things and you could, you didn't have to be perfect because you were moving regardless.”* There was also comfort in the logistics of the centrally located intervention and the time of day it was offered.

“This was to me so much easier than going to the [campus recreation center] and signing up for classes with students. Yeah. I don't know. I don't like working out with 17-year-olds.”

“And sometimes I have a small child at home and this class is the only exercise I get. Even though I try to be active at home, it's very hot outside. Going for a walk around the neighborhood sometimes just isn't reasonable, so this is sometimes it.”

“… time, I’m usually like, once I leave here, my life just gets chaotic and before I know it's time to go to sleep. And so this was so convenient that it was during work hours.”

#### Connectedness

The group-based design of the intervention made participants feel a social connection. Most expressed having a positive experience due to the social group dynamic of the dance fitness classes.

“I’ve never done a dance class before and I didn't feel out of place at all. I didn't feel like they were professional dancers and I was just here. It felt like we were all a big group”.

“I think I’ve gotten closer with some of the coworkers I worked with because of this class, to know people a little better.”

“It was just something about being in this group setting and with the people that I was with that was encouraging and motivating.”

### Actual changes

Participation in the intervention led to the reporting of changes in behavior and associated perceived health outcomes. For example, one shared how their energy levels were influenced and how this changed caffeine consumption behaviors.

“I could stay up longer. And when I told my friends, like, are you serious? Like you’re just working out for like, how much was like a month and a half or something like that? And I started, like, showing that, oh, I can stay up later, because at night I was just crashing and I kept going. And it helped me with caffeine intake. Actually, I lowered it because it was coming here, and then I had to push my lunch later. So my afternoon coffee became more of like, hey, do you need one? Or just like go with water?”

There were other reports of positive health changes in concentration and feelings of physical fitness. *“It helped to improve my concentration, focus. And then I noticed that when I walked to and from the car like to from my car to the building I’m not like out of breath.”*

Others who felt physical changes from the intervention reported behavioral changes associated with PA. One woman expressed how the intervention routine eliminated an exercise barrier for her. *“I got in a routine of, like, packing my clothes in the morning. That was a big shift. And not having that excuse or barrier.”* Another shared an increase in PA behaviors. *“I’ve started going on a walk with a friend.”*

Another woman explained how the intervention changed how she viewed time as a barrier to exercising. Having experienced a 30-min exercise class with WEALTH shifted how she believed she could incorporate exercise into her workday. *“I also used to think I couldn't do 30 min in the middle of the day, like it seemed like I can’t take this long break, but just coming over here, I mean it seriously only took about 45 total minutes.”*

These reported changes positively influenced participants’ intention to continue being physically active post-intervention. Overall, they felt they were more likely to continue exercising because of the positive exercise experiences associated with WEALTH.

## Discussion

All women will experience menopause and the subsequent adverse health effects caused by physiological changes. Public health professionals advise women to engage in a physically active life to prevent adverse health outcomes associated with menopause and aging (Grindler and Santoro, 2015; Hulteen et al., 2023). Our study provides a first step to understanding intervention strategies and lived experiences that may be useful to improve the protective PA behaviors and health of women before menopause occurs. We discuss this understanding alongside practical implications. A consensus from our findings is that PA programs that provide 30-min durations of fun, group-based, women-only modalities may grant the consistency with exercise women report a desire to have in their lives and reframe exercise as an accessible lifestyle behavior.

### Predetermined experiences

We founded our consensus on the understanding that women have life experiences that shape their current relationship with exercise. Most women in our study had a negative relationship with exercise. Numerous factors serve as motivators to engage in exercise. Some external motivators, such as weight loss, hinder sustainable exercise participation. Other internally driven motivators, such as relieving stress, facilitate behavioral commitment to exercise (Hagger and Chatzisarantis, 2007). We saw the pattern evolve where women held a negative relationship with exercise. However, after participating in a positively perceived exercise experience, they felt a shift in this relationship. There appears to be promise in shifting women’s negative perceptions of exercise toward a positive relationship that fosters sustained PA engagement throughout the lifespan. This is especially noteworthy when considering findings showing individuals who hold negative memories of childhood exercise are likelier to have poor exercise adherence (Daalen, 2005; Digelidis et al., 2003; Beltrán-Carrillo et al., 2012). Therefore, intervention designs that validate and shift the negative exercise relationships women embody could help women engage in protective PA behaviors for better health.

Participants’ experiences showed that predetermined exercise attitudes were connected to their desire for consistency. More specifically, we saw that one’s damaging relationship with exercise was connected to their actual ability to have consistent exercise engagement. “*I do find out sometimes like I have a negative relationship with exercise where I’ll judge myself if it's been too long. It's really hard to get back into it.”* This quote contextualizes how a woman’s damaging relationship with exercise creates a cycle of judgment and eventual behavioral barriers. Still, there is a craving for movement that women expressed with their desire to have exercise be a consistent aspect of their lives. Women’s experiential exercise beliefs are connected to their exercise behaviors and inherent health outcomes. Such findings align with health behavior theories that explain how intention, the most proximal determinant of behavior, is indeed influenced by the beliefs one has towards the behavior, which are influenced by one’s personhood (Fishbein, 2008).

In line with women expressing a desire for exercise consistency, we noted a belief that prompts this desire. When discussing this idea of consistency, we noted that women wanted to make exercise easier. *“Just make it a treat and it's going to be easier to do.”* Such a perception provides insight into the female experience with exercise as a behavioral concept that is difficult, and this perception of difficulty is connected to behavioral consistency. We question whether the barrier to sustained PA across womanhood is because exercise itself is physically challenging or that being able to exercise consistently is the actual difficulty. Health behavioral intention is theorized to be directly influenced by how difficult one perceives a behavior to be (Fishbein, 2008; Fishbein, 2011). While women may perceive exercise as difficult in the physical sense, which is arguably rooted in the gender role typing of exercise, our findings showcase that the priority barrier is the perceived difficulty in achieving exercise consistency. With women collectively expressing this hunger for consistent exercise in their lives and other shared attitudes toward exercise being linked back to this desire, there appears to be promise in addressing the issue of consistency. As such, we suggest interventions focus on influencing women to feel more capable of doing consistent exercise rather than solely physically capable of performing exercise.

### Intervention design factors

If women are charged to champion regular exercise rather than exercise performance, it could be easier to choose movement modalities that are sustainable for their lifestyle needs and preferences. Many forms of PA have been shown to improve health outcomes (Noetel et al., 2024). However, little work has considered the activities tailored to women’s needs and interests (Anderson et al., 2023; Exercise Movement and Dance Partnership, 2022; Segar et al., 2002). Results of meta-analytic reviews suggest that women are more likely to sustain their involvement in PA if they are allowed to exercise with others in social or group-based settings rather than on their own (Bullard et al., 2019; Burke et al., 2006). Choice in the modality of PA engagement is a gendered phenomenon, with multiple differences in women’s and men’s exercise preferences and behaviors (Reading and Jessica, 2022; Uffelen et al., 2017).

The design of a group-based dance fitness intervention influenced the sub-theme of fun that women reported. We noted that modality and group nature positively influenced women’s exercise experiences and behaviors. We propose that enjoyment is a factor that not only positively influences women’s exercise behaviors but also reshapes negative, predetermined thoughts and experiences with exercise. The design features of the intervention that prompted participants’ talk of fun were the social nature and how the movement made them feel. Women may find group-based and accessible movement, such as dance fitness, the most enjoyable form of exercise. These feelings of enjoyment can prompt women to exercise more consistently through the associated positive experiences. This is supported by other findings where women reported a greater likelihood to exercise and positive health outcomes when they believed they would or indeed had fun exercising (Hadfield et al., 2022; Stanley and Jennifer, 2010). For example, women dominate dance fitness programs, which are also one of the top preferred forms of group exercise modalities among women (Exercise Movement and Dance Partnership, 2022). Moreover, meta-analytic evidence showed that dance interventions for at least 6 weeks significantly improve psychological health outcomes, equivalent to other forms of structured exercise and PA (Yan et al., 2024).

The favorability toward a movement modality within a group setting garnered positive beliefs and experiences toward exercise among participants. This aligns with other researchers who found that women who participated in group-based exercise were more likely to sustain exercise over time due to feelings of accountability and social wellness (Golaszewski et al., 2022). With women’s PA levels decreasing with age (Varma et al., 2017) and women in our study reporting positive attitudes toward the group nature of WEALTH, the social element of exercise may be a key factor in positively impacting premenopausal women’s PA levels and, subsequently, through menopause.

We also saw that objective factors informed participants’ views and spoke more broadly to women’s exercise relationships. Among adults, perceived lack of time is one of the barriers most cited to participating in regular, health-enhancing PA (Trost et al., 2002). The time barrier was noted when participants discussed the ease of attending WEALTH during the workday because of its central location and the time of day it was offered. They viewed WEALTH as addressing this barrier and allowing them to exercise more efficiently, which spoke to the theme of desired exercise consistency. We argue that the barrier of perceived lack of time relates to women’s lack of exercise consistency, as expressed in our study. Time as a barrier to women’s exercise engagement is rooted in sociocultural issues. For women in the workforce, the time demands of work, family, and social responsibilities make participation even more challenging as they balance competing interests (Government of Canada, 2015; Bureau of Labor Statistics, 2024). Recent estimates show that only 17.6%–28.7% of women meet the PA guidelines proven to prevent disease and promote health (Elgaddal et al., 2020), which we suggest is, in essence, due to women being overburdened at home, in the workforce, and in society. We urge health and fitness practitioners to challenge the normative script that an hour or more is needed to make exercise “count” or “worthy.” The most recent PA guidelines indicate that any bout of PA counts toward achieving the guidelines and reaping the health benefits (U.S. Department of Health and Human Services, 2018). Women would benefit from a cultural norm within exercise where movement can be incorporated into shorter durations, and even in the workplace. Moreover, employers may want to consider having centrally located spaces for employees to be active and socially connected. Researchers have found that such programming positively influenced employees’ intention to exercise during the workday (Hadfield et al., 2020). Worksite PA interventions that employ these practices could show an investment in healthy behaviors and positive health outcomes throughout adulthood. With older adult women leaving the workforce in more significant numbers due to menopausal symptoms and a perceived lack of support (Whiteley et al., 2013), a cultural shift in women’s exercise may retain critical numbers of women in the workforce.

A significant design characteristic of WEALTH was its women-only design. Participants connected the women-only design to their feelings of comfort, which we noted must play a role in women’s reshaping of negative experiences and ease of exercise consistency. With men historically being more physically active than women worldwide (The Lancet Public Health, 2019), gender is essential to explore in PA research. Human movement is indeed gendered, with normative scripts influencing behaviors, which, in the context of PA, impacts health. We can more easily understand how the gendered phenomenon of human movement informs PA beliefs, such as those found in our study, and these beliefs inform how women move throughout adulthood, which impacts their health status. Spaces that offer comfort to women seeking exercise consistency could increase PA levels across womanhood rather than continue the ever-decreasing PA levels women are experiencing worldwide.

### Behavior and health changes

Participants reported changes in behavior and associated perceived health outcomes. Our main consensus holds the idea that perceptual changes inform behavior change. Our findings of participants experiencing health behavior changes, such as increased daily PA, were connected to the pattern of altered perceptions of exercise. Women described how participating in WEALTH prompted them to view exercise as something they could successfully do while working full-time and upholding all their interests and responsibilities. The experience of this shift in perception created space to change other health behaviors and feel health outcomes, such as having more energy throughout the day. The WEALTH trial primarily assessed participants’ PA levels, yet supplemental outcomes were noted through our qualitative analysis, which quantitative methodologies did not account for. The narrative inquiry we used bolstered the notion that PA interventions can have various impacts outside of the primary assessment of PA. Therefore, researchers may consider broader health interests worthy of exploration, as these secondary outcomes may speak more about women’s unique needs and experiences and their PA behaviors. Also, future research can further explore the impact of secondary outcomes to assess the nuanced relationship women have with exercise beliefs, behaviors, and experiences to inform women’s health intervention designs better.

### Limitations

We acknowledge some limitations. These were focus groups conducted at a single time point for each group, and we did not check findings from our analyses with participants. The intervention instructor was a part of the research team and conducted the analyses. We acknowledge that the involvement of the intervention instructor as a member of the research team presents potential risks of bias. To address potential issues with such dynamics, we used common validity strategies in qualitative research (Carspecken, 2013; Dennis, 2020). Specifically, the research team engaged in regular peer debriefing sessions during data collection and analysis, involving researchers who were independent of the instructor role, to critically examine emerging themes and interpretations. We also conducted negative case analysis by actively seeking and examining data that contradicted initial patterns, ensuring that our findings were not solely based on confirmatory evidence. We did not find any dissenting voices through this process. Additionally, we employed a strip analysis technique, involving cross-checking data segments to verify consistency and reduce undue influence. Regarding coding procedures, the instructor initially conducted coding independently; then, a second researcher independently coded a subset of the same transcripts without influence from the instructor’s codes. The codes were compared, discrepancies discussed, and a consensus reached to enhance reliability. This process was repeated at regular intervals throughout the analysis. These steps aimed to ensure the transparency and trustworthiness of our findings despite the dual role of the instructor while addressing the risk of bias.

Also, we acknowledge that the title of Table 1, “Descriptive characteristics of study sample (N = 28),” pertains to the larger quantitative component of this research. The qualitative focus group of focus in this manuscript included 11 participants. Due to the scope of the current study, demographic data for the focus group participants are not available beyond what has already been reported, and this is acknowledged as a limitation.

### Practical application suggestions

We acknowledge that our study’s sample consisted of a small (n = 11), specific group of full-time employees at a single university in the southern United States. As such, the findings should be interpreted with care and are not intended to be broadly generalizable. While our results offer preliminary insights into women-centered PA interventions for premenopausal women within this particular context, the feasibility and effectiveness of these strategies may vary across different populations, settings, and cultural backgrounds. Consequently, we recommend that future research examine these approaches in larger, more diverse samples to better understand their broader applicability. The practical suggestions presented should be viewed as preliminary guidance that warrants further validation rather than prescriptive solutions for all populations.

Overall, our study’s findings offer valuable guidance for designing women-centered PA interventions tailored to premenopausal women. Programs that emphasize 30-min, enjoyable, group-based activities, such as dance fitness, held during the workday can foster consistency by making exercise feel accessible, social, and fun. These interventions should focus on reshaping negative perceptions of exercise and promoting a sense of capability, rather than performance, to support long-term adherence. Creating women-only spaces and incorporating social support elements can help alleviate gender-related barriers and enhance comfort. Additionally, integrating flexible, shorter activity bouts into workplace or community settings can address perceived time constraints and normalize PA as an achievable and enjoyable lifestyle habit. While these strategies are particularly relevant for premenopausal women, they hold potential for broader application across different life stages, emphasizing the importance of tailored, gender-sensitive approaches to promote sustained PA and improved health outcomes for women.

## Conclusion

PA programs that provide 30-min durations of fun, group-based, women-only modalities during the workday may provide the consistency with exercise that women report a desire to have in their lives. Such movement opportunities can reframe exercise as a positive and accessible lifestyle behavior for women and promote sustainable PA levels across the lifespan. Through our analysis aimed at understanding factors that inform premenopausal women’s PA beliefs, behaviors, and experiences, we recognized how the unique lived experiences of being a woman inform women’s relationship with exercise. This embodied experience informs the complex nature of women’s exercise and, we argue, showcases the gender disparities in PA levels and health. Such complexity is worthy of consideration when designing women’s PA interventions.

## Data Availability

The raw data supporting the conclusions of this article will be made available by the authors, without undue reservation.
